# Pyoderma Gangrenosum in a Patient with Crohn’s Disease Treated with Adalimumab: A Case-Based Review and Systematic Review of the Current Literature

**DOI:** 10.3390/clinpract15030057

**Published:** 2025-03-11

**Authors:** Fotios S. Fousekis, Konstantinos Mpakogiannis, Emmanouil Karampinis, Ioanna Nefeli Mastorogianni, Dimitrios K. Christodoulou, Marina Papoutsaki, Evanthia Zampeli, Konstantinos H. Katsanos

**Affiliations:** 1 Department of Gastroenterology and Hepatology, University Hospital of Ioannina, University of Ioannina, 45110 Ioannina, Greece; kostismpakogiannis@gmail.com (K.M.); nefelimastorogianni@gmail.com (I.N.M.); dchristodoulou@gmail.com (D.K.C.); khkostas@hotmail.com (K.H.K.); 2 Second Dermatology Department, School of Health Sciences, Aristotle University of Thessaloniki, 54124 Thessaloniki, Greece; ekarampinis@uth.gr; 3 Department of Dermatology, Faculty of Medicine, School of Health Sciences, University General Hospital of Larissa, University of Thessaly, 41110 Larissa, Greece; 4 1st Departament of Dermatology-Venereology, Faculty of Medicine, National and Kapodistrian University of Athens, “A. Sygros” Hospital for Skin and Venereal Diseases, 16121 Athens, Greece; marinapapoutsaki@hotmail.com; 5 Department of Gastroenterology, Alexandra General Hospital, 11528 Athens, Greece; evazamb@gmail.com

**Keywords:** pyoderma gangrenosum, Crohn’s disease, adalimumab, inflammatory bowel disease, extra-intestinal manifestations

## Abstract

**Background:** Pyoderma gangrenosum (PG) is a rare inflammatory cutaneous disorder that frequently occurs in association with systemic diseases such as inflammatory bowel disease (IBD). This case report describes a 23-year-old female with Crohn’s disease (CD) who developed PG and was successfully treated with adalimumab. The objective of this study is to present the clinical course, treatment approach, and outcomes while reviewing the existing literature on the efficacy of adalimumab in PG management. **Methods:** A case report is presented, detailing clinical presentation, diagnostic evaluation, and treatment strategy. Additionally, a systematic review was conducted using PubMed to assess studies on adalimumab in PG, focusing on treatment response, remission rates, and adverse effects. **Results:** The patient presented with ulcerative lesions on her lower extremities and sacroiliitis. After corticosteroid therapy, adalimumab was initiated, leading to significant ulcer healing, reduced back pain, and CD remission. The systematic review identified seven studies on adalimumab in PG. Findings suggest that adalimumab is effective in steroid-refractory cases, with remission achieved in a significant proportion of patients. The most common adverse effects were infections, but overall, adalimumab showed a favorable safety profile. **Conclusions:** This case highlights the importance of early diagnosis and multidisciplinary management of PG in CD patients. Adalimumab appears to be a promising therapeutic option, particularly for steroid-resistant PG, though further research is needed to establish standardized treatment protocols.

## 1. Introduction

Pyoderma gangrenosum (PG) is a rare, painful, ulcerative, cutaneous condition with distinctive clinical characteristics [1]. The term “pyoderma gangrenosum” was introduced by Brunsting in 1930, referring to a case-series of patients suffering either ulcerative colitis (UC) or chronic purulent pleurisy. The word “pyoderma” reflects the condition’s pus-producing aspect, while “gangrenosum” highlights its progressive necrotic nature [2]. PG generally appears in individuals between their 30s and 60s, with a nearly equal prevalence among men and women [3]. A PG lesion starts as a tender nodule, plaque, or sterile pustule, which over days enlarges into a sharply defined ulcer with purplish borders and surrounding redness. Pain is a common feature. The skin and underlying tissue may become necrotic, resulting in a fragile wound bed with hemorrhagic or purulent discharge that can extend to the muscle [2,4]. As the lesions heal, they often leave behind sieve-like scars [5]. Lesions are usually multiple and recurrent, with 25–50% triggered by trauma, a reaction known as pathergy [6]. PG lesions most commonly affects the lower extremities; however, any anatomical site can be involved, such as the face, trunk, and genital regions [7].

There are currently no specific serologic or pathological criteria for diagnosing PG. Thus, PG remains a diagnosis of exclusion, relying on the rapid onset of painful ulcers with distinct borders and the exclusion of other causes. Furthermore, a history of systemic diseases, characteristic scarring patterns, a positive response to immunosuppressive treatment, and evidence of pathergy support the diagnosis [8]. Unfortunately, the diagnosis of PG is often established only after multiple ineffective treatment attempts, which include surgical debridement and antibiotics. Lesions typically worsen when treated as infections and can deteriorate further due to pathergy following surgical procedures [9]. Diagnosing pyoderma gangrenosum mainly depends on clinical evaluation, as biopsies do not show any pathognomonic diagnostic characteristics. However, a biopsy is crucial to exclude other conditions such as malignancies, infections, cutaneous vasculitis, or infections. Differential diagnoses should include a variety of bacterial, mycobacterial, and fungal infections (like sporotrichosis, aspergillosis, and cryptococcosis), as well as parasitic and viral infections such as herpes simplex [10]. Other conditions to consider involve primary cutaneous tumors, metastatic skin cancer, skin lymphomas [11], and rare disorders like mycosis fungoides bullosa and Langerhans cell histiocytosis [12]. Hematological disorders such as sickle cell disease and cryoglobulinemia may also present similarly. In addition, causes may include autoimmune conditions (e.g., systemic lupus erythematosus, Behçet’s disease, Wegener granulomatosis, and polyarteritis nodosa) and vascular issues like livedoid vasculopathy, venous stasis ulcers, or small-vessel arterial occlusions [13]. Furthermore, some ulcers are associated with cryoglobulinemia and antiphospholipid syndrome [14]. It is also essential to differentiate pyoderma gangrenosum from conditions like drug-induced lupus and factitious disorders, including Munchausen syndrome, as these may mimic its presentation. Finally, drug reactions or external injuries should also be considered as potential causes [15].

Due to the diagnostic complexity of PG, researchers and experts have collaborated to develop a diagnostic algorithm. Three diagnostic criteria have been proposed: the Su and Delphi consensus, and the PARACELSUS score [16]. All these diagnostic criteria appear to be beneficial in clinical practice, but comparative studies have shown the PARACELSUS score to have the highest sensitivity [17].

PG is linked to underlying systemic diseases in at least 50% of cases. Commonly associated conditions include inflammatory bowel disease (IBD), arthritis, and hematologic disorders. PG may be diagnosed either after the underlying disease or as its initial manifestation [18,19]. Therefore, it is crucial to assess each patient with PG for potential comorbidities in order to manage these effectively to improve the overall prognosis. In particular, PG appears to be an unusual but severe extra-intestinal manifestation (EIM) of IBD [19]. A recent meta-analysis of fourteen studies included 1057 patients with IBD and 26 cases of PG from a Louisville cohort. Among 61,695 IBD patients, there were 379 cases of PG, with an incidence ranging from 0.4% to 2.6% in individual studies [20]. In this case-based review, we discuss a case of PG in a patient with CD who was treated with adalimumab, analyzing pathophysiology and management options.

Dysregulation of the immune system plays a crucial role in the pathophysiology of PG. In PG lesions, abnormal neutrophils and T-cells have been identified, with inflammatory mediators such as IL-1β, IL-8, IL-17, and TNF-α significantly contributing to the condition [21,22]. Trauma can induce or worsen PG through the pathergy phenomenon, which triggers the release of IL-36 and autoantigens from damaged keratinocytes [23]. This release, in turn, increases the expression of IL-8 and IL-17. IL-17, produced by Th17 cells, is known to be involved in inflammatory conditions like psoriasis and is also found at elevated levels in PG skin lesions [24,25]. Furthermore, Th17 cells are overrepresented in these lesions, while regulatory T cells (Treg) and their associated cytokines, such as IL-10, are present in lower levels [26]. In addition, TNF triggers the expression of adhesion molecules on endothelial cells, which helps neutrophils migrate to inflamed areas, a hallmark of pyogenic granuloma (PG) autoinflammation [27,28]. Specifically, TNF priming boosts the interactions between FcgammaR and immune complexes, enhancing the binding of Mac-1 to ICAM-1. This process effectively retains neutrophils at sites of inflammation [28].

## 2. Case Presentation

**Figure 1:**
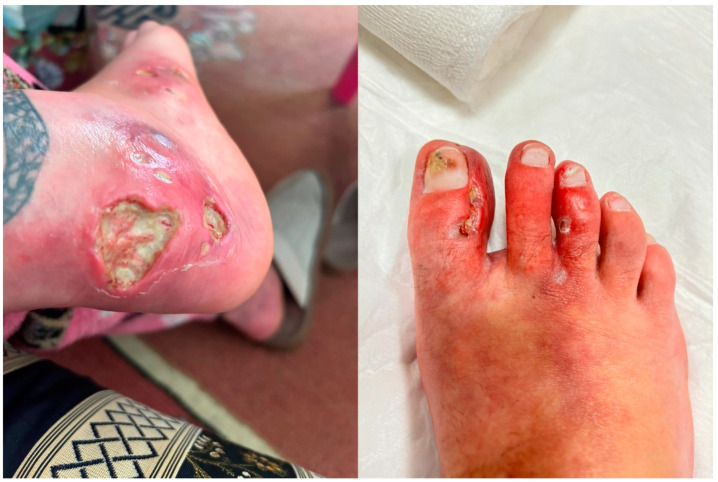
Clinical presentation of pyoderma gangrenosum lesions on the lower extremity and toes. The left picture shows multiple deep ulcerative lesions with irregular margins on the heel, characterised by erythematous surrounding skin and central purulent exudate. The right picture shows inflammatory changes on the toes with erythema, pustules, and ulceration consistent with pyoderma gangrenosum.

A 23-year-old female patient with a history of CD, specifically diagnosed ileitis approximately two years earlier, was admitted to the hospital due to the progression of ulcerative lesions on her lower extremities. Purulent discharge and persistent erythematous swelling in her left shin accompanied these lesions (Figure 1). Her medical history extends over seven years, beginning with the appearance of subcutaneous edematous lesions on her lower limbs, which gradually developed into ulcerative wounds. Additionally, the patient reported experiencing lower back pain, particularly in the mornings, over the past few months. Due to CD, she has been undergoing treatment with budesonide.

In the workup of her skin ulcers, cultures were taken from the lesions, and a skin biopsy was performed. She was tested for sarcoidosis, with serum ACE levels returning negative. Additionally, a Doppler ultrasound of her lower extremities was undergone to rule out vascular pathology, and no abnormalities were detected. Screening for the initiation of a biological agent was performed concurrently. Cultures from the cutaneous lesions were negative, while histopathological analysis revealed neutrophilic infiltrates in the epidermis, pseudoepitheliomatous hyperplasia, and lymphoplasmacytic infiltration in the dermis, with occasional mitotic activity. A pathergy test was performed and the result was positive. Given her reported back pain, an MRI of the sacroiliac joints was performed, revealing findings consistent with sacroiliitis. Based on her diagnosis of axial spondyloarthritis and the suspected diagnosis of pyoderma gangrenosum, the patient received corticosteroids with a rapid tapering regimen, followed by the introduction of adalimumab. The choice of adalimumab was supported by evidence of its efficacy in managing both pyoderma gangrenosum and axial spondyloarthritis. 

**Figure 2:**
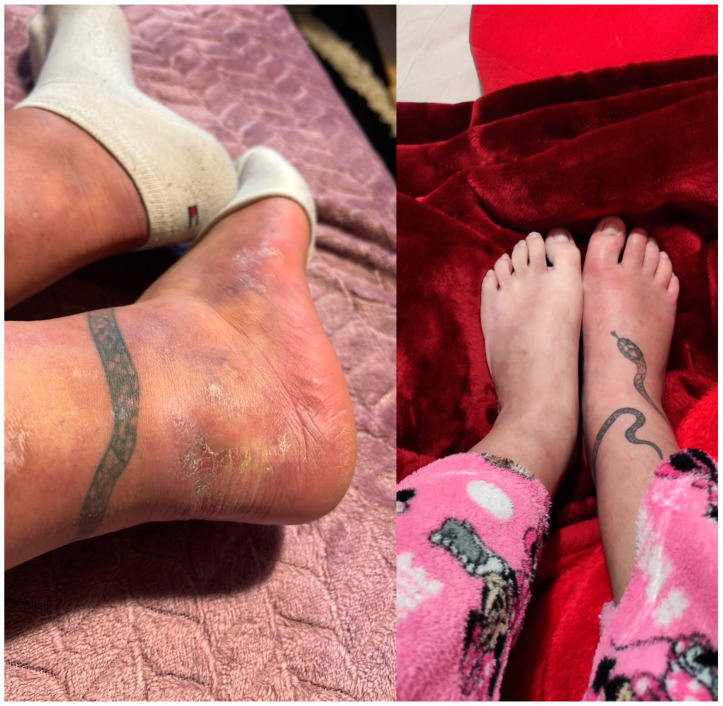
Clinical improvement of pyoderma gangrenosum lesions after treatment with adalimumab. The left image shows the heel with marked resolution of the ulcerative lesions, now replaced by post-inflammatory hyperpigmentation and areas of mild scarring, with no active signs of purulent exudate or inflammation. The right image shows the toes which appear largely healed with minimal residual erythema and no evidence of active ulceration or pustular lesions.

Three months later, during a follow-up visit at the outpatient clinic, the patient had discontinued corticosteroids and was maintained on adalimumab at a dose of 40 mg every two weeks. There was significant regression of the ulcerative lesions (Figure 2), and the patient reported marked clinical improvement in her lower back pain. Additionally, she did not report any gastrointestinal symptoms, indicating clinical remission of CD. Therefore, in our case, adalimumab has been demonstrated to be an effective therapeutic option for the management of both pyoderma gangrenosum and axial spondyloarthritis, while maintaining CD in remission.

## 3. Material and Methods

**Figure 3:**
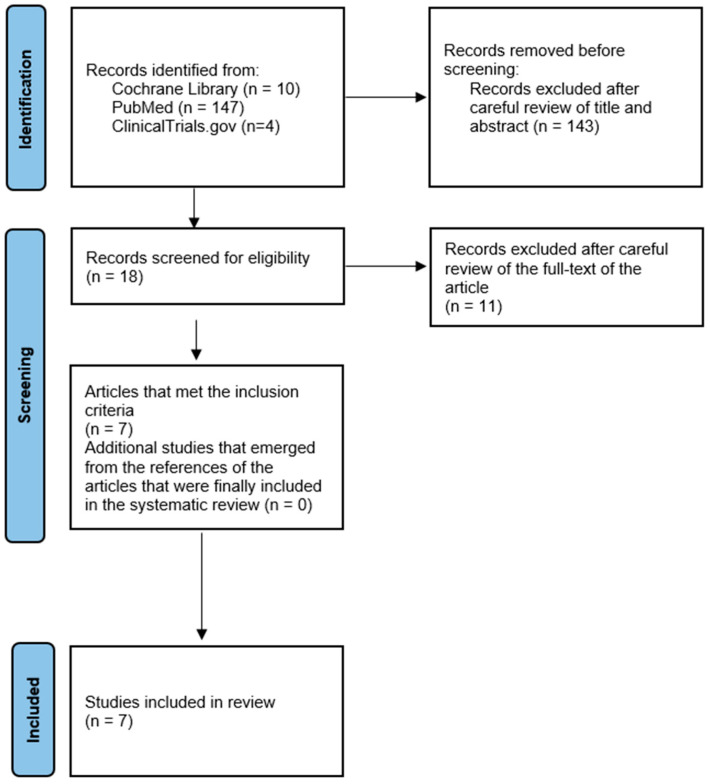
PRISMA flowchart.

There is growing evidence that adalimumab is an effective option for the treatment of pyoderma gangrenosum, even in steroid-refractory cases (61). In order to find the most relevant studies on this topic, we conducted a search of the PubMed database up to 23 December 2024. We used the following search string: “adalimumab” AND “pyoderma gangrenosum”. Inclusion criteria were randomized clinical trials, and case series and observational studies that included at least 3 patients with pyoderma gangrenosum treated with adalimumab. We excluded case reports and studies with two or fewer patients as well as articles not written in English. Additionally, the reference lists of all selected articles were carefully reviewed to identify any further relevant studies. Two independent investigators (F.S.F and K.M) conducted the data extraction process independently. This review was conducted in accordance with the PRISMA 2020; however, it has not been registered. The search strategy is illustrated in the PRISMA flowchart (Figure 3).

## 4. Results

The total search yielded 161 articles, of which 143 were excluded after reviewing the title and abstract. Subsequently, 11 articles were excluded after careful examination of the full text of the article. Specifically, three articles were excluded due to lack of data [29,30,31,32]. In accordance with the exclusion criteria, an additional article was excluded on the basis that it concerned only one patient with pyoderma gangrenosum [33]. For a similar reason, three articles were excluded because they contained only two cases of patients with pyoderma gangrenosum who received adalimumab [34,35,36]. Furthermore, two articles were excluded from the review as they were provisional results of a study published in 2022 that included its final results in our systematic review [36,37,38]. Finally, the exclusion of one study was necessitated by its ongoing recruitment phase (NCT04750213), while another study (NCT03311464) corresponds to the PubMed article [38] that was included in the final review. 

Seven studies in total were included in our systematic review and summarized in Table 1. A 52-week phase 3 open-label study involving Japanese patients with active pyoderma gangrenosum ulcers demonstrated the significant efficacy of adalimumab. By week 26, 54.5% of patients had achieved complete skin re-epithelialisation (PGAR 100), with continued improvements observed during the extension period, where 66.7% had achieved PGAR 100 by week 52. Furthermore, notable improvements were observed in pain and quality of life over the course of the study. Adverse events were documented in 18 patients, including four cases of serious infections. However, the treatment was generally well tolerated [38]. In a case series of four patients (three with CD and one with UC), clinical improvement was observed within a median of 11 days, with complete lesion healing occurring within a median of 34 days. No severe adverse effects were reported, indicating a favorable safety profile [39]. In an interim analysis of a post-marketing study on adalimumab for pyoderma gangrenosum (PG), 42.9%, 36.8%, and 50.0% of patients achieved a Physician Global Assessment score of 0/1 at weeks 12, 26, and 52, respectively. It is noteworthy that systemic steroids were used by 70.3% of patients prior to and 56.8% during the course of treatment. Furthermore, adverse drug reactions were observed in 18.9% of patients, with infections documented in 13.5% and serious infections in 10.8% [40]. In a retrospective, dual-center cohort study evaluating treatment outcomes for patients with pyoderma gangrenosum treated with biologics, complete remission or significant improvement was observed in 57.1% (16/28) of those receiving adalimumab. The treatment also resulted in an average wound size reduction of 38.1 cm². Among these 28 patients, adverse events reported included infection (3 cases), seizure (1 case), and leukocytoclastic vasculitis (1 case). However, it should be noted that no data are available regarding causation of these adverse events [41]. Furthermore, cure of pyoderma gangrenosum was achieved in 10 out of 10 patients using adalimumab in a retrospective study from Spain. The median healing period was 4–8 weeks. No adverse events were documented [42]. In a case series comprising three patients with diverse underlying conditions (CD, PAPA syndrome, and PASH syndrome), only one patient (with CD) exhibited a positive response to adalimumab. No adverse effects were observed during the follow-up period, which ranged from one month to three years [43]. In a case-series of three patients diagnosed with CD who developed refractory peristomal PG, topical steroid lotion, prednisone, and adalimumab or a combination of these agents were used in all three patients. All ulcers healed within 6 months [44].

These findings (Table 1) indicate that adalimumab may be an effective treatment option for pyoderma gangrenosum, including patients with underlying inflammatory conditions and IBD. However, the potential for serious adverse events underscores the importance of careful patient selection and monitoring during treatment. Further randomized controlled trials are required to confirm these observations.

## 5. Discussion

Treating PG, especially with CD, requires a multidisciplinary approach with collaboration between dermatologists and gastroenterologists; PG management focuses on suppressing the immune response and controlling inflammation. For milder cases or as supplementary treatment, high-potency topical corticosteroids and calcineurin inhibitors (such as tacrolimus) are frequently used. They seem to have similar efficacy and are well tolerated [45]. Although topical corticosteroids are effective even at lower potencies, they are not without risks. Potential adverse effects include skin atrophy, telangiectasia, striae, purpura, tachyphylaxis, steroid-induced acne, and ulceration [46]. Conversely, the main side effect of topical calcineurin inhibitors is a burning sensation at the site of application, which may be alleviated by refrigerating the medication before use [47,48]. Intralesional steroid injections can also be helpful, particularly for smaller ulcers or localized conditions [49]. Nevertheless, it is important to use this treatment cautiously to prevent trigger pathergy and to avoid disrupting wound healing. Intralesional methotrexate has demonstrated some clinical benefits as well [50]. In addition, various other topical medications have been used to treat PG, each with differing success rates. Cromolyn sodium (1–4%) has been effective as monotherapy or in combination [51], while benzoyl peroxide (20%) accelerated re-epithelialization [52]. Additionally, aminosalicylic acid (10%), phenytoin (2%), and nicotine (0.5%) have also been tested, with several cases achieving complete healing [52]. Becaplermin, a recombinant human platelet-derived growth factor, promoted granulation tissue formation, and timolol enhanced wound healing when combined with other treatments [53,54]. Furthermore, proper wound care is essential to prevent infection and manage pain. Wound management involves gentle cleansing without aggressive debridement, limited use of topical antibacterial agents, and maintenance of a moist environment to promote epithelial cell migration [55].

Corticosteroids are considered as the first-line treatment for PG due to their rapid anti-inflammatory effect [56]. They primarily act by inhibiting NF-κB and reducing pro-inflammatory cytokines [57]. Systemic corticosteroids are administered at a dose of 0.5–1 mg/kg/day, which results in a clinical response in approximately 40–50% of cases [58]. However, the rates of complete remission may vary significantly based on the severity of PG and any associated diseases [59]. Once healing occurs, the corticosteroid dosage is gradually tapered over several months. In cases of severe or multi-lesional PG, corticosteroids are often combined with immunosuppressants like cyclosporine to enhance treatment outcomes [60]. For a more immediate response, pulse therapy with intravenous methylprednisolone can be used to initiate or improve treatment effectiveness (56). In our case, we administered corticosteroids for a short duration of approximately one month to minimize side effects. This treatment was also combined with adalimumab, leading to a significant clinical improvement. It is important to note that a major challenge to the use of adalimumab in PG is its limited availability to patients who do not have CD or another approved indication. In many regions, adalimumab is only covered for certain autoimmune diseases, making it difficult for PG patients without these conditions to access treatment. This can lead to delays in treatment or the need to use less effective alternatives.

Cyclosporine, a calcineurin inhibitor that suppresses cytokine transcription, is also considered as a first-line treatment for PG [61]. The STOP GAP trial compared oral prednisolone (0.75 mg/kg/day) and cyclosporine (4 mg/kg/day), finding no difference in healing speed, treatment response, or quality of life. Nearly half of the patients healed within six months, but about a third experienced recurrence after around 582 days. Both treatments had similar rates of adverse effects [62]. The choice between prednisolone and cyclosporine depends on patient comorbidities; cyclosporine is often preferred for large ulcers or in cases where prednisolone poses higher risks [63]. Cyclosporine has also shown success in treating multi-lesional PG, sometimes in combination with corticosteroids. In IBD, cyclosporine is indicated for inducing remission in acute severe UC. Therefore, given the patient’s history of CD, we ultimately chose to use corticosteroids [64].

Recent studies have explored the efficacy of other biologic therapies in the treatment of PG, particularly in cases that are refractory to other treatments. These include infliximab, another TNF-α inhibitor [65]; ustekinumab, an IL-12/IL-23 inhibitor commonly used in psoriasis and CD [66]; and secukinumab [67], an IL-17 inhibitor. Furthermore, IL-1 blockade with anakinra has been attempted in refractory PG [68], and rituximab, a CD20 monoclonal antibody targeting B cells, has also been reported in PG treatment, particularly in cases with an autoimmune background [69]. While these biologics present promising options for steroid-refractory PG, further randomized controlled trials are needed to determine their efficacy and optimal use in clinical practice.

The disease has a chronic course with episodes of exacerbation, which renders the long-term prognosis challenging. An early diagnosis and subsequent treatment are crucial for a favorable prognosis. The prompt administration of anti-inflammatory medications, such as corticosteroids or immunosuppressants, can effectively halt disease progression and promote healing. Furthermore, it is essential to direct attention towards the management of underlying conditions, because PG is not just a cutaneous disorder, but also a cutaneous manifestation of systemic inflammation that significantly impacts the patient [70]. Concerning IBD, it appears to be a favorable prognostic factor. According to cross-sectional analysis, patients with underlying hematologic malignancy/dyscrasia and vasculitis had a fourfold increased risk of in-hospital mortality, while there was an increase in the utilization of healthcare resources [71]. It was also noted that the prognosis of PG may be significantly impacted by treatment delays or inadequate management. When left untreated or when patients are refractory to conventional therapies, PG can progress into a severely debilitating condition, leading to extensive ulceration, tissue destruction, and even loss of function in affected areas. Furthermore, non-adherence to treatment can accelerate disease progression, increasing the risk of complications such as infections, chronic wounds and, in severe cases, amputation [72].

## 6. Study Limitations

First, the literature search was limited to the PubMed, Cochrane, and ClinicalTrials.gov databases potentially excluding relevant studies from other sources like Embase. Additionally, the small number of included studies reflects the rarity of PG, which limits the generalizability of findings. Variability in treatment regimens and outcome measures among studies may also introduce bias. Lastly, while adalimumab appears to be effective for steroid-refractory PG, the risk of serious adverse events underscores the need for standardized treatment protocols and further randomized trials.

## 7. Conclusions

Adalimumab, a TNF-α inhibitor, has shown efficacy in the treatment of both PG and CD, as demonstrated in this case, highlighting its potential as an effective therapy for patients with both disorders. PG remains a diagnostic challenge, often leading to delayed recognition and inappropriate initial therapies that may exacerbate disease. The rarity of PG in CD and the variability of its presentation challenge clinicians in establishing standardized treatment protocols. In addition, biologics such as adalimumab, while promising, carry the risk of adverse effects, including serious infections, which require careful patient monitoring. Larger, long-term randomized controlled trials are essential to better define the efficacy, safety, and optimal treatment strategies for adalimumab in PG management. Future research should focus on elucidating the common immunological pathways between CD and PG in order to refine current therapeutic strategies. A multidisciplinary approach involving gastroenterologists and dermatologists is essential for the timely diagnosis and effective management of PG in CD patients.

## Figures and Tables

**Table 1:** Key studies on the efficacy and safety of adalimumab in the treatment of pyoderma gangrenosum.

Study Design	Number of Patients	Underlying Disease	Follow-Up Period	Outcome	Adverse Events	Study (Ref.)
Prospective (52-week phase 3 open-label study)	22 patients	4 Pts UC 3 Pts RA	26 weeks 52 weeks	At 26 weeks: 12 of 22 pts achieve 100% reduction in the PG area. At 52 weeks: 6 of 9 pts achieve 100% reduction in the PG area.	Serious AEs in 4 Pts (18%), including one each of anemia, bacterial arthritis (which led to the discontinuation of the study drug), cataract, and pain due to PG.	Yamasaki K et al. [37]
Retrospective	28 patients	Not available	67.4 months	complete remission or improvement in 57.1% (16/28) of patients	Infection (3) Seizure (1) Leukocytoclastic vasculitis (1)	Herberger K et al. [41]
Retrospective	4 patients	3 Pts CD 1 Pt UC	5–40 months	Clinical improvement in 11 days (median, range 5–15), with complete PG lesion healing in 34 days (median, range 15–60).	No severe adverse effects noticed.	Cariñanos I et al. [39]
Retrospective	32 patients	8 Pts UC 3 Pts CD 3 RA	Up to 52 weeks	PGA score of 0/1 for total lesions At wk 12: 42.9% At wk 26: 36.8% At wk 52: 50.0%	10.8% develop serious infections as ADR	Yamamoto T et al. [36]
Retrospective	3 patients	1 Pt CD 1 Pt PAPA syndrome 1 Pt PASH syndrome	1 month to 3 years	1 of 3 pts respond to ADA (CD patient)	None	George A et al. [43]
Retrospective	10 patients	10 Pts IBD	N/A	10 of 10 patients respond to ADA Timing healing: 4–8 weeks	None	Argüelles-Arias F et al. [42]
Retrospective	3 patients	3 Pts CD	6 months	3 of 3 patients respond to ADA	None	Toyoda T et al. [44]

ADA: adalimumab, ADR: adverse drug reaction, PG: pyoderma gangrenosum, Pt: Patient, RA: rheumatoid arthritis, UC: ulcerative colitis, PGA score: Physician Global Assessment score (total lesions).

## Data Availability

The data described in this study are available upon request from the corresponding author.

## References

[1] Maverakis E., Marzano A.V., Le S.T., Callen J.P., Brüggen M.-C., Guenova E., Dissemond J., Shinkai K., Langan S.M. (2020). Pyoderma Gangrenosum. Nat. Rev. Dis. Primers.

[2] Ahronowitz I., Harp J., Shinkai K. (2012). Etiology and Management of Pyoderma Gangrenosum. Am. J. Clin. Dermatol..

[3] Langan S.M., Groves R.W., Card T.R., Gulliford M.C. (2012). Incidence, Mortality, and Disease Associations of Pyoderma Gangrenosum in the United Kingdom: A Retrospective Cohort Study. J. Investig. Dermatol..

[4] Su W.P.D., Davis M.D.P., Weenig R.H., Powell F.C., Perry H.O. (2004). Pyoderma Gangrenosum: Clinicopathologic Correlation and Proposed Diagnostic Criteria. Int. J. Dermatol..

[5] Vidal D., Puig L., Gilaberte M., Alomar A. (2004). Review of 26 Cases of Classical Pyoderma Gangrenosum: Clinical and Therapeutic Features. J. Dermatol. Treat..

[6] Binus A.M., Qureshi A.A., Li V.W., Winterfield L.S. (2011). Pyoderma Gangrenosum: A Retrospective Review of Patient Characteristics, Comorbidities and Therapy in 103 Patients. Br. J. Dermatol..

[7] Barbe M., Batra A., Golding S., Hammond O., Higgins J.C., O’Connor A., Vlahovic T.C. (2021). Pyoderma Gangrenosum. Clin. Podiatr. Med. Surg..

[8] Maverakis E., Ma C., Shinkai K., Fiorentino D., Callen J.P., Wollina U., Marzano A.V., Wallach D., Kim K., Schadt C. (2018). Diagnostic Criteria of Ulcerative Pyoderma Gangrenosum. JAMA Dermatol..

[9] Jain A.G., Sharbatji M., Afzal A., Afridi S.M., Gordon D. (2019). Pyoderma Gangrenosum in the Absence of Any Underlying Predisposing Condition: A Diagnostic Dilemma. Cureus.

[10] Alonso-León T., Hernández-Ramírez H.H., Fonte-Avalos V., Toussaint-Caire S., Vega-Memije M.E., Lozano-Platonoff A. (2020). The Great Imitator with No Diagnostic Test: Pyoderma Gangrenosum. Int. Wound J..

[11] Aseni P., Di Sandro S., Mihaylov P., Lamperti L., Carlis L.G. (2008). De Atypical Presentation of Pioderma Gangrenosum Complicating Ulcerative Colitis: Rapid Disappearance with Methylprednisolone. World J. Gastroenterol..

[12] Norris J.F.B., Marshall T.L., Byrne J.P.H. (1984). Histiocytosis X in an Adult Mimicking Pyoderma Gangrenosum*. Clin. Exp. Dermatol..

[13] Bennett M.L., Jackson J.M., Jorizzo J.L., Fleischer A.B., White W.L., Callen J.P. (2000). Pyoderma Gangrenosum A Comparison of Typical and Atypical Forms with an Emphasis on Time to Remission. Case Review of 86 Patients from 2 Institutions. Medicine.

[14] Ashchyan H.J., Nelson C.A., Stephen S., James W.D., Micheletti R.G., Rosenbach M. (2018). Neutrophilic Dermatoses. J. Am. Acad. Dermatol..

[15] Weenig R.H., Davis M.D.P., Dahl P.R., Su W.P.D. (2002). Skin Ulcers Misdiagnosed as Pyoderma Gangrenosum. N. Engl. J. Med..

[16] Dissemond J., Marzano A.V., Hampton P.J., Ortega-Loayza A.G. (2023). Pyoderma Gangrenosum: Treatment Options. Drugs.

[17] Haag C., Hansen T., Hajar T., Latour E., Keller J., Shinkai K., Ortega-Loayza A.G. (2021). Comparison of Three Diagnostic Frameworks for Pyoderma Gangrenosum. J. Investig. Dermatol..

[18] Lebrun D., Robbins A., Hentzien M., Toquet S., Plee J., Durlach A., Bouaziz J.-D., Bani-Sadr F., Servettaz A. (2018). Two Case Reports of Pyoderma Gangrenosum and Systemic Lupus Erythematosus. Medicine.

[19] He R., Zhao S., Cui M., Chen Y., Ma J., Li J., Wang X. (2023). Cutaneous Manifestations of Inflammatory Bowel Disease: Basic Characteristics, Therapy, and Potential Pathophysiological Associations. Front. Immunol..

[20] States V., O’Brien S., Rai J.P., Roberts H.L., Paas M., Feagins K., Pierce E.J., Baumgartner R.N., Galandiuk S. (2020). Pyoderma Gangrenosum in Inflammatory Bowel Disease: A Systematic Review and Meta-Analysis. Dig. Dis. Sci..

[21] Takeuchi F., Streilein R.D., Hall R.P. (2003). Increased E-selectin, IL-8 and IL-10 Gene Expression in Human Skin after Minimal Trauma. Exp. Dermatol..

[22] Marzano A.V., Ortega-Loayza A.G., Heath M., Morse D., Genovese G., Cugno M. (2019). Mechanisms of Inflammation in Neutrophil-Mediated Skin Diseases. Front. Immunol..

[23] Lowes M.A., Russell C.B., Martin D.A., Towne J.E., Krueger J.G. (2013). The IL-23/T17 Pathogenic Axis in Psoriasis Is Amplified by Keratinocyte Responses. Trends Immunol..

[24] Caproni M., Antiga E., Volpi W., Verdelli A., Venegoni L., Quaglino P., Fabbri P., Marzano A.V. (2015). The Treg/Th17 Cell Ratio Is Reduced in the Skin Lesions of Patients with Pyoderma Gangrenosum. Br. J. Dermatol..

[25] Gameiro A., Pereira N., Cardoso J.C., Gonçalo M. (2015). Pyoderma Gangrenosum: Challenges and Solutions. Clin. Cosmet. Investig. Dermatol..

[26] Jeucken K.C.M., Koning J.J., Mebius R.E., Tas S.W. (2019). The Role of Endothelial Cells and TNF-Receptor Superfamily Members in Lymphoid Organogenesis and Function During Health and Inflammation. Front. Immunol..

[27] Lauterbach M., O’Donnell P., Asano K., Mayadas T.N. (2008). Role of TNF Priming and Adhesion Molecules in Neutrophil Recruitment to Intravascular Immune Complexes. J. Leucoc. Biol..

[28] Łyko M., Ryguła A., Kowalski M., Karska J., Jankowska-Konsur A. (2024). The Pathophysiology and Treatment of Pyoderma Gangrenosum—Current Options and New Perspectives. Int. J. Mol. Sci..

[29] Louis E.J., Reinisch W., Schwartz D.A., Löfberg R., Robinson A.M., Berg S., Wang A.W., Maa J., Huang B., Pappalardo B. (2018). Adalimumab Reduces Extraintestinal Manifestations in Patients with Crohn’s Disease: A Pooled Analysis of 11 Clinical Studies. Adv. Ther..

[30] Travis S., Feagan B.G., Peyrin-Biroulet L., Panaccione R., Danese S., Lazar A., Robinson A.M., Petersson J., Pappalardo B.L., Bereswill M. (2017). Effect of Adalimumab on Clinical Outcomes and Health-Related Quality of Life Among Patients With Ulcerative Colitis in a Clinical Practice Setting: Results from InspirADA. J. Crohns Colitis.

[31] Watanabe M., Hibi T., Lomax K.G., Paulson S.K., Chao J., Alam M.S., Camez A. (2012). Adalimumab for the Induction and Maintenance of Clinical Remission in Japanese Patients with Crohn’s Disease. J. Crohns Colitis.

[32] Cossio M.-L., Genois A., Jantchou P., Hatami A., Deslandres C., McCuaig C. (2020). Skin Manifestations in Pediatric Patients Treated With a TNF-Alpha Inhibitor for Inflammatory Bowel Disease: A Retrospective Study. J. Cutan. Med. Surg..

[33] Barreiro-de-Acosta M., Lorenzo A., Domínguez-Muñoz J.E. (2012). Efficacy of Adalimumab for the Treatment of Extraintestinal Manifestations of Crohn’s Disease. Rev. Esp. Enfermedades Dig..

[34] Nakayama Y., Akeda T., Iida S., Habe K., Yokota N., Matsushima Y., Nakai Y., Kondo M., Yamanaka K. (2021). Whether to Maintain or Strengthen the Treatment for Pyoderma Gangrenosum Ulcerative Type May Depend on the Response after Two to Four-week Treatment Intervention: The Outcome of Three Cases with Details Clinical Course. Clin. Case Rep..

[35] Vacas A.S., Torre A.C., Bollea-Garlatti M.L., Warley F., Galimberti R.L. (2017). Pyoderma Gangrenosum: Clinical Characteristics, Associated Diseases, and Responses to Treatment in a Retrospective Cohort Study of 31 Patients. Int. J. Dermatol..

[36] Yamamoto T. (2021). An Update on Adalimumab for Pyoderma Gangrenosum. Drugs Today.

[37] Yamasaki K., Yamanaka K., Zhao Y., Iwano S., Takei K., Suzuki K., Yamamoto T. (2020). Adalimumab in Japanese Patients with Active Ulcers of Pyoderma Gangrenosum: Twenty-six-week Phase 3 Open-label Study. J. Dermatol..

[38] Yamasaki K., Yamanaka K., Zhao Y., Iwano S., Takei K., Suzuki K., Yamamoto T. (2022). Adalimumab in Japanese Patients with Active Ulcers of Pyoderma Gangrenosum: Final Analysis of a 52-week Phase 3 Open-label Study. J. Dermatol..

[39] Cariñanos I., Acosta M.B.-D., Domènech E. (2011). Adalimumab for Pyoderma Gangrenosum Associated with Inflammatory Bowel Disease. Inflamm. Bowel Dis..

[40] Yamamoto T., Yamanaka K., Yamasaki K., Isaji H., Matsubara N., Hozawa H., Kawakami T. (2024). Real-world Safety and Effectiveness of Adalimumab in Patients with Pyoderma Gangrenosum: Interim Analysis of a Post-marketing Observational Study in Japan. J. Dermatol..

[41] Herberger K., Dissemond J., Brüggestrat S., Sorbe C., Augustin M. (2019). Biologics and Immunoglobulins in the Treatment of Pyoderma Gangrenosum—Analysis of 52 Patients. J. Dtsch. Dermatol. Ges..

[42] Argüelles-Arias F., Castro-Laria L., Lobatón T., Aguas-Peris M., Rojas-Feria M., Barreiro-de Acosta M., Soto-Escribano P., Calvo-Moya M., Ginard-Vicens D., Chaparro-Sánchez M. (2013). Characteristics and Treatment of Pyoderma Gangrenosum in Inflammatory Bowel Disease. Dig. Dis. Sci..

[43] George A., Sathishkumar D., Mathew L., Gupta A., Chiramel M.J., Singh V., Thomas M. (2024). Clinicopathological Profile of Pyoderma Gangrenosum: A 10-Year Retrospective Study from a Tertiary Care Center in South India. Indian Dermatol. Online J..

[44] Toyoda T., Mitsuyama S., Nagao E., Abe F., Kimura M., Seido Y., Higuchi T. (2021). Topical Management of Peristomal Pyoderma Gangrenosum. J. Wound Ostomy Cont. Nurs..

[45] Donnelly H., Boffa M.J. (2024). Topical Treatment of Pyoderma Gangrenosum: A Systematic Review. Indian J. Dermatol. Venereol. Leprol..

[46] Hengge U.R., Ruzicka T., Schwartz R.A., Cork M.J. (2006). Adverse Effects of Topical Glucocorticosteroids. J. Am. Acad. Dermatol..

[47] Ghislain P.-D., De Decker I., Marot L., Lachapelle J.-M. (2004). Efficacy and Systemic Absorption of Topical Tacrolimus Used in Pyoderma Gangrenosum. Br. J. Dermatol..

[48] Cecchi R., Pavesi M., Bartoli L., Brunetti L. (2012). Successful Treatment of Localized Pyoderma Gangrenosum with Topical Pimecrolimus. J. Cutan. Med. Surg..

[49] Goldstein F., Krain R., Thornton J.J. (1985). Intralesional Steroid Therapy of Pyoderma Gangrenosum. J. Clin. Gastroenterol..

[50] Baltazar D., Haag C., Gupta A.S., Marzano A.M., Ortega Loayza A.G. (2019). A Comprehensive Review of Local Pharmacologic Therapy for Pyoderma Gangrenosum. Wounds.

[51] Tamir A., Landau M., Brenner S. (1996). Topical Treatment with 1% Sodium Cromoglycate in Pyoderma Gangrenosum. Dermatology.

[52] Chow R.K.P., Ho V.C. (1996). Treatment of Pyoderma Gangrenosum. J. Am. Acad. Dermatol..

[53] Zhao X., Gu H., Xu Z., Zhang Q., Lv X., Zheng X., Yang Y. (2014). Efficacy of Topical Recombinant Human Platelet-Derived Growth Factor for Treatment of Diabetic Lower-Extremity Ulcers: Systematic Review and Meta-Analysis. Metabolism.

[54] Liu D.Y., Fischer R., Fraga G., Aires D.J. (2014). Collagenase Ointment and Topical Timolol Gel for Treating Idiopathic Pyoderma Gangrenosum. J. Am. Acad. Dermatol..

[55] Croitoru D., Naderi-Azad S., Sachdeva M., Piguet V., Alavi A. (2020). A Wound Care Specialist’s Approach to Pyoderma Gangrenosum. Adv. Wound Care.

[56] Maronese C.A., Pimentel M.A., Li M.M., Genovese G., Ortega-Loayza A.G., Marzano A.V. (2022). Pyoderma Gangrenosum: An Updated Literature Review on Established and Emerging Pharmacological Treatments. Am. J. Clin. Dermatol..

[57] Cruz-Topete D., Cidlowski J.A. (2015). One Hormone, Two Actions: Anti- and Pro-Inflammatory Effects of Glucocorticoids. Neuroimmunomodulation.

[58] Kolios A.G.A., Gübeli A., Meier B., Maul J.-T., Kündig T., Nilsson J., Hafner J., Guenova E., Kerl K., Anliker M. (2017). Clinical Disease Patterns in a Regional Swiss Cohort of 34 Pyoderma Gangrenosum Patients. Dermatology.

[59] Yamamoto T., Yamasaki K., Yamanaka K., Komine M., Kawakami T., Yamamoto O., Kanekura T., Higuchi T., Takahashi T., Matsushima Y. (2023). Clinical Guidance of Pyoderma Gangrenosum 2022. J. Dermatol..

[60] Tan M.G., Tolkachjov S.N. (2024). Treatment of Pyoderma Gangrenosum. Dermatol. Clin..

[61] Ormerod A.D., Thomas K.S., Craig F.E., Mitchell E., Greenlaw N., Norrie J., Mason J.M., Walton S., Johnston G.A., Williams H.C. (2015). Comparison of the Two Most Commonly Used Treatments for Pyoderma Gangrenosum: Results of the STOP GAP Randomised Controlled Trial. BMJ.

[62] Mason J.M., Thomas K.S., Ormerod A.D., Craig F.E., Mitchell E., Norrie J., Williams H.C. (2017). Ciclosporin Compared with Prednisolone Therapy for Patients with Pyoderma Gangrenosum: Cost-Effectiveness Analysis of the STOP GAP Trial. Br. J. Dermatol..

[63] Marzano A.V., Trevisan V., Lazzari R., Crosti C. (2011). Pyoderma Gangrenosum: Study of 21 Patients and Proposal of a ‘Clinicotherapeutic’ Classification. J. Dermatol. Treat..

[64] García M.J., Riestra S., Amiot A., Julsgaard M., García de la Filia I., Calafat M., Aguas M., de la Peña L., Roig C., Caballol B. (2024). Effectiveness and Safety of a Third-line Rescue Treatment for Acute Severe Ulcerative Colitis Refractory to Infliximab or Ciclosporin (REASUC Study). Aliment. Pharmacol. Ther..

[65] Zaman M., Martinez R., Mayur O., Montoya M., Serwald G., McNichol M.C., McGee J.S. (2024). Use of biologic therapies in the management of pyoderma gangrenosum: A systematic review. Arch. Dermatol. Res..

[66] Miklusiak K., Miklusiak K., Kaczmarczyk O., Cibor D., Zwolińska-Wcisło M. (2023). Ustekinumab in the treatment of acute disseminated pyoderma gangrenosum in a patient with Crohn’s disease. Dermatol. Rep..

[67] McPhie M.L., Kirchhof M.G. (2020). Pyoderma gangrenosum treated with secukinumab: A case report. SAGE Open Med. Case Rep..

[68] O’Connor C., Gallagher C., Hollywood A., Paul L., O’Connel M. (2021). Anakinra for recalcitrant pyoderma gangrenosum. Clin. Exp. Dermatol..

[69] DaCunha M., Siscos S., Downing M., Tarantino I., Hall J. (2019). Pyoderma gangrenosum controlled with rituximab. JAAD Case Rep..

[70] Ben Abdallah H., Bech R., Fogh K., Olesen A.B., Vestergaard C. (2021). Comorbidities, Mortality and Survival in Patients with Pyoderma Gangrenosum: A Danish Nationwide Registry-nested Case–Control Study. Br. J. Dermatol..

[71] Kaffenberger B.H., Hinton A., Krishna S.G. (2018). The Impact of Underlying Disease State on Outcomes in Patients with Pyoderma Gangrenosum: A National Survey. J. Am. Acad. Dermatol..

[72] Iliescu C., Popa L., Mihai M., Popescu M.N., Beiu C. (2024). Pyoderma Gangrenosum: The Impact of Treatment Non-adherence on Disease Progression. Cureus.

